# Genetic propensity for obesity, socioeconomic position, and trajectories of body mass index in older adults

**DOI:** 10.1038/s41598-021-99332-7

**Published:** 2021-10-13

**Authors:** Kristiane Tommerup, Olesya Ajnakina, Andrew Steptoe

**Affiliations:** 1 Department of Behavioural Science and Health, Institute of Epidemiology and Health Care, University College London, 1-19 Torrington Place, Gower Street, London, UK; 2 Department of Biostatistics and Health Informatics, Institute of Psychiatry, Psychology and Neuroscience, King’s College London, London, UK

**Keywords:** Genetic interaction, Risk factors

## Abstract

Identifying how socioeconomic positioning and genetic factors interact in the development of obesity is imperative for population-level obesity prevention strategies. The current study investigated whether social positioning, either independently or through interaction with a polygenic score for Body Mass Index (BMI-PGS), influences BMI trajectories across older adulthood. Data were analysed from 7,183 individuals from the English Longitudinal Study of Aging (ELSA). Interactions between the BMI-PGS and; lower educational attainment, self-perceived social status (SSS), and income, on BMI trajectories over 12 years across older adulthood were investigated through linear mixed effects models. Lower educational attainment, SSS and income were each associated with a higher baseline BMI for women, but not for men. There were interaction effects between BMI-PGS and social positioning such that men aged > 65 with a lower educational attainment (β = 0.62; 95%CI 0.00 – 1.24, p < 0.05), men aged ≤ 65 of a lower income (β = − 0.72, 95%CI − 1.21 - − 0.23, p < 0.01) and women aged ≤ 65 of lower SSS (β = − 1.41; 95%CI − 2.46 – 0.36, p < 0.01) showed stronger associations between the BMI-PGS and baseline BMI. There were few associations between markers of socioeconomic position and rate of change in BMI over the follow-up period. In sum, lower socioeconomic positioning showed adverse associations with women’s BMI in older adulthood. Moreover, the expression of the BMI-PGS, or extent to which it translates to a higher BMI, was subtly influenced by socioeconomic standing in both women and in men.

## Introduction

The prevalence of obesity, defined in adulthood by a body mass index (BMI) ≥ 30^1^, is associated with numerous adverse health implications in older age. These include an increased risk for diabetes^2^, cardiovascular diseases^3^, hypertension and even mortality^4^. This relationship is anticipated to further increase as the general population continues to age, with associated costs of obesity being estimated to reach £49.9 billion per year by 2050^5^.

The distribution of obesity is unequal across socioeconomic positions (SEP)^6^, with rates being higher among those with lower education^7^, income^8^ or subjective social class^9^. Obesity rates demonstrate a strong gradient in the UK as result of a greater exposure to the obesogenic environment and more limited opportunities for adequate nutrition and physical activity across the lifespan^10^. While education, self-perceived social standing (SSS) and income are correlated, they reflect different aspects of SES at older ages^11^. Education is typically completed in early life and shapes occupational trajectories; while income is an indicator of economic resources that influences opportunities for mobility, food choice, and access to exercise facilities relevant to adiposity in later life^12^. Moreover, there is evidence to suggest that women’s BMI in adulthood appears to be more adversely affected by lower socioeconomic settings than in men^6^. However, it is less well understood how BMI may be differentially influenced by specific markers of SEP between the sexes, potentially highlighting more specific and fruitful targets for obesity prevention efforts in older adulthood.

Nonetheless, obesity has a strong genetic basis, with an estimated heritability ranging from ~ 40 to 70%^13^. To uncover the molecular mechanisms underlying BMI, genome-wide association studies (GWAS) have been successful in identifying hundreds of significant loci associated with BMI, which together are shown to have a substantial additive influence on BMI^14^. These GWAS have led to the development of the polygenic score approach, which represents an aggregate measure of polygenic risk for BMI by exploiting all loci associated with a higher BMI^15^. Polygenic approaches also offer novel means to uncover the potentially modifying effect of the social environment on the expression of genetic risk towards a higher BMI. A more favourable socioeconomic environment may attenuate the expression of genetic risk for BMI, while a less favourable environment may enhance the expression of genetic risk. Hence, the same genetic risk may result in higher BMI for populations living in lower socioeconomic conditions; a so called gene-environment interaction (GxE)^16^. A recent study from Barcellos et al. found a significant gene-by-education interaction within the large UK Biobank Sample^17^. Moreover, Frank et al. demonstrated the presence of GxE in relation to income, highlighting the potential for SEP measures outside of educational attainment to attenuate genetic risk towards BMI^18^. However, the support for GxE in relation to obesity remains inconsistent^19–21^. Moreover, GxE studies of BMI have primarily focused on educational attainment, and therefore alternative aspects of socioeconomic positioning remain unexplored.

The current study used a large population-representative cohort of older adults to investigate whether a higher genetic load of multiple risk alleles for BMI (a BMI-PGS score) was associated with BMI at baseline (~ 64 years of age) and rate of change in BMI over a 12-year follow-up period in older adults. We tested interactions between the BMI-PGS score and educational attainment, income, and SSS in relation to BMI at baseline and change in BMI over the follow-up period. We were also interested in how such interactions may operate differently in mid-to late adulthood (50–65 years of age) and late adulthood (≥ 65) as BMI trajectories increase in mid-to-late adulthood and then begin to decrease in late adulthood^22^. Hence the dichotomisation utilised in the current study was derived in line with demonstrated upwards trends of BMI trajectories in mid-to late adulthood (< 65) and downwards trends across older adulthood (≥ 65)^22^.

We hypothesised that adults with a larger genetic susceptibility (BMI-PGS) would be at greater risk for accelerated increases in BMI over the 12-year period. Secondly, we hypothesised that the BMI-PGS would be more strongly associated with BMI at baseline and steeper BMI trajectories for individuals of a lower educational attainment, income or SSS; a GxE interaction.

## Method

### Sample

Data were drawn from the English Longitudinal Study of Aging (ELSA), a nationally representative survey of English adults aged 50 years or older^23^. Measures of socioeconomic position and covariates were taken from wave 2 (2004–2005) for the core sample (82%), or waves 4 (2008–2009) and 6 (2012–2013) for the respective refreshment samples (18%). Baseline measures of BMI were taken from wave 2 (2004–2005) for participants who provided blood samples for genotyping at wave 2 (77%), or wave 4 for those who provided blood samples at wave 4 (23%). Follow-up BMI measures were obtained from waves 6 (2012–2013) and 8 (2016–2017). Ethical approval for each ELSA wave was granted by the National Research Ethics Service (London Multicentre Research Ethics Committee). All participants gave informed consent, and all experiments were performed in accordance with relevant guidelines and regulations.

### Study variables

#### Body mass index (BMI)

BMI was calculated using standard formulae (weight in kilograms/height in square meters)^22^. Here, height and weight were measured during the nurse visit. Weight was measured using Tanita electronic scales to measure body weight without shoes and in light clothing. Height was determined by Stadiometer using the Frankfort plane on a ground level. At Wave 8, BMI was calculated using height measurements obtained from Wave 6, as no height measurements were obtained in 2016–2017.

### Measures of socioeconomic position

#### Educational attainment

Educational Attainment was measured through self-reported highest educational qualification. Respondents were asked to self-report their highest educational qualification or attainment using computer-assisted interviewing, from a list response option including: degree level qualifications, teaching qualifications, nursing qualifications, A-levels or higher school certificate, O-level qualifications, GCSE level graded, NVQ qualifications, apprenticeship level, other qualifications, or none. Responses were derived into three categories: (1) Higher Qualification (undergraduate or postgraduate degree level), (2) Secondary Qualification (A/O or GSCE level or equivalent), (3) Primary Qualification (Below A/O/GSCE or no qualification).

#### Subjective social status (SSS)

SSS was measured through the MacArthur Scale of Subjective Social Status^24^. This measure presents a drawing of a ladder with 10 rungs to respondents, representing where people stand in society, the higher up representing those with the most money, education, and jobs. Respondents were asked to place a single “X” on this ladder to rank their social standing, producing a score from 1 to 10, with 10 being the highest SSS. These raw scores were derived into tertiles: (1) Top Tertile, (2) Middle Tertile, (3) Lowest Tertile of SSS.

#### Income

Income was measured through self-reported household equivalised income, adjusted for variation in household size. Income was calculated from detailed assessments of a full range of earned and unearned sources of income. Earned sources of income included employment income, self-employment income, state pension income, and other benefit income; while unearned sources on income included income from assets, investments and financial transfers^25^. The income variable was divided into tertile to represent the groups of individuals with (1) High, (2) Intermediate and (3) Low Tertiles of Income.

#### Covariates

Demographic covariates included marital status (not currently married vs currently married) derived from a single item asking participants to disclose their current legal marital status as; single, married, remarried, legally separated, divorced, or widowed. Behavioural covariates included smoking status (not-current smoker vs current smoker) derived from one item asking participant whether they smoke cigarettes at all nowadays (yes vs no). Secondly, physical activity level (sedentary or low activity at least once a week, moderate activity at least once a week, vigorous activity at least once a week) was also included in the models, and derived through three separate items reporting on the frequency of either vigorous, moderate, or mildly energetic sports and activities; more than once a week, once a week, one to three times a month, or hardly ever or never^26^. These responses were categorised according to their highest level of activity reported at least once a week. Health related covariates included the presence of a longstanding illness (illness reported vs. no illness reported) reported through a single item; Do you have any long-standing illness, disability, or infirmity? (yes/no) and depressive symptomatology (current depressive symptoms vs no current depressive symptoms). Depressive symptoms were measured with an 8-item version of the Centre for Epidemiologic Studies Depression Scale^27^, which has comparable psychometric properties to the full 20-item scale; a score ≥ 4 was used to define participants with severe depressive symptoms^28^. Lastly, genetic ancestry measured with principal components (see below), was included as a covariate (four principal components) to account for any ancestry differences in genetic structures that could bias our results^29^.

### Genetic data

The genome-wide genotyping was performed at University College London Genomics in 2013–2014 using the Illumina HumanOmni2.5 BeadChips (HumanOmni2.5-4v1, HumanOmni2.5-8v1.3), which measures ~ 2.5 million markers that capture the genomic variation down to 2.5% minor allele frequency (MAF). Samples were removed based on call rate (< 0.99), suspected non-European ancestry as identified though principal components analysis and self-identification, heterozygosity, and relatedness. Specifically, to investigate population structure, principal components analysis (PCA)^29^ in PLINK 1.9^30^ was conducted^21^. An inspection of PCA highlighted the presence of ancestral admixture in the 65 individuals. We removed these outliers and re-calculated PCs using the updated samples; here, top 10 principal components were retained to account for any ancestry differences in genetic structures that could bias results^31,32^.

Duplicated samples and cryptic relatedness between each pair of participants was evaluated using pairwise genome-wide estimates of three coefficients corresponding to the probabilities of sharing 0, 1 or 2 alleles between two individuals that are identical by descent^33^. We used the method of moments for estimating the identical by descent (IBD) probabilities^34^ implemented in PLINK^30^ 1.9. IBD were estimated using autosomal SNPs where IBD = 1 highlights presence of duplicates or monozygotic twins, IBD = 0.5 shows that first-degree relatives are present in the sample, IBD = 0.25 and IBD = 0.125 highlights presence of second-degree and third-degree relatives, respectively^35^. We identified individuals with an IBD value of > 0.2 and excluded one of each pair at random^36^. Single Nucleotide polymorphisms (SNPs) were excluded if they were non-autosomal, the minor allele frequency was < 0.01%, if more than 2% of genotype data were missing and if the Hardy–Weinberg Equilibrium P < 10^−4^.

#### Polygenic score (PGS)

To calculate PGS for BMI (BMI-PGS), we used summary statistics reported by the Genetic Investigation of Anthropometric Traits (GIANT) consortium (2018)^14^. BMI-PGS were calculated as a weighted sum of the allele dosages, summing over the markers abiding by the *p*-value threshold (*P*_T_) (i.e., 0.001, 0.01, 0.05, 0.1, 0.3, and 1) weighted according to the strength of effect estimate were summed in a continuous score using PRSice. Using information on sample size (*n*), total number of independent markers in genotyping panel (*m*) and lower and upper *P*values to select markers into polygenic score we estimated the predictive accuracy (*R*^2^) *P*_T_ = 0.001 (*m* = 255,091), *P*_T_ = 0.01 (*m* = 114,862), *P*_T_ = 0.05 (*m* = 62,583), *P*_T_ = 0.1 (*m* = 194,940), *P*_T_ = 0.3 (*m* = 412,954), and *P*_T_ = 1 (*m* = 798,737) we estimated a predictive power of each PGS using Avenge me package implemented in R^37,38^.. Consequently, we estimated predictive accuracy for each PGS at *P*_T_ = 0.001 (*R*^2^ = 0.001, *P* = 0.004), *P*_T_ = 0.01 (*R*^2^ = 0.001, *P* = 0.035), *P*_T_ = 0.1 (*R*^2^ = 0.03, *P* = 3.83 × 10^–6^), *P*_T_ = 0.05 (*R*^2^ = 0.002, *P* = 1.31 × 10^–5^), *P*_T_ = 0.03 (*R*^2^ = 0.001, *P* = 0.003) and *P*_T_ = 0.1 (*R*^2^ = 0.001, *P* = 0.014) had sufficient, as indicated by significant *P *values, predictive accuracy to be employed in the analyses. As previously a large comparative study showed that a PGS at *p *value thresholds *P*_T_ = 1 was the ultimate PGS to use in longitudinal studies^39,40^_,_ we utilised PGS that was based on *P*_T_ = 1 assuming all genetic markers contribute to trait development.

### Statistical analyses

To assess the interplay between BMI-PGS with socioeconomic position on BMI values at baseline and across the 12-year follow up period, we employed linear mixed effect models (LMMs) with maximum likelihood estimation^41^. LMMs with maximum likelihood estimation maximise the use of longitudinal data, adjust for the correlation between repeated measures, weight estimates for missing data between waves, and increase statistical power and precision^41^. Using Akaike Information Criterion and Bayesian Information Criterion, a quadratic model allowing for random intercepts and slopes was deemed most appropriate for our analyses. To test whether variation in BMI across SEP influenced the model results, heteroscedascity assumptions was examined, and where heteroscedascity was present, models used robust standard errors, using the vce(robust) command in STATA, relaxing the assumption that standard errors carry identical and equal distributions^42^. Interactions between BMI-PGS and all three measures of socioeconomic position were investigated using multiplicative models. Each analysis was stratified by gender and age group (i.e., < 65 years old vs > 65 years old). We used a significance level of 0.05 (two-tailed) for all analyses. All analyses were conducted in STATA release 16 (STATA Corp LP, USA)^43^.

#### Sensitivity analyses

In the sensitivity analyses we repeated all analyses as described above but with missing values imputed for both socioeconomic position (educational attainment, SSS, and income) and all covariate measures using MissForest in RStudio version^44^ 3.6.2.6.

## Results

### Sample characteristics

The total sample consisted of 7183 ELSA participants for whom the quality-controlled genome-wide genotyping and BMI during the follow-up were available; of these 46% (N = 3304) were men and 54% (N = 3879) were women. The baseline mean age for men was 64.40 (standard deviation (SD) = 9.15) and for women was 64.35 (SD = 9.56). A larger proportion of men (74.88%) than women (56.17%) reported a longstanding illness (x^2^ = 6.11, P = 0.011); whereas a larger proportion of women (34.37%) than men (21.35%) showed elevated depressive symptoms (x^2^ = 148.59, P < 0.001). Men and women differed further in terms of marital status, level of physical activity, income, and educational attainment all reported at baseline (Table 1).

**Table 1:** Baseline sample characteristics of ELSA participants.

Sample characteristics	Men (n = 3304)	Women (n = 3878)	Test statistics		
	N(%)/mean (SD)	N(%)/mean (SD)	t/x^2^	*Df*	P value
Age (years)	64.40 (9.15)	64.35 (9.56)	− 0.31	7180	0.75
**Source of baseline BMI**					
Wave 2	2461 (74.48)	2937 (75.73)	1.35	1	0.25
Wave 4	753 (22.79)	841 (21.68)			
Current smoker	512 (15.57)	653 (16.89)	2.26	1	0.13
Not married	780 (23.61)	1466 (37.79)	167.06	1	< 0.001
**Income**					
High	1244 (38.71)	1207 (32.15)	49.81	2	< 0.001
Moderate	997 (31.02)	1130 (30.10)			
Low	973 (30.27)	1417 (37.75)			
**Highest educational attainment**					
Higher qualification	1117 (35.80)	861 (25.04)	90.02	2	< 0.001
Secondary qualification	812 (26.03)	1040 (30.24)			
Primary qualification	1191 (38.17)	1538 (44.72)			
**Subjective social status**					
Top Tertile	637 (20.33)	585 (15.90)	24.35	2	< 0.001
Middle Tertile	2215 (70.68)	2780 (75.54)			
Lower Tertile	282 (9.00)	315 (8.56)			
Longstanding Illness present	1758 (74.88)	2177 (56.17)	6.11	1	0.01
Poor self-reported health	1772 (23.37)	1938 (24.18)	0.64	1	0.43
**Physical activity**					
Sedentary	532 (16.13)	803 (20.75)	35.63	2	< 0.001
Moderate activity	1578 (47.85)	1878 (48.53)			
Vigorous activity	1188 (36.02)	1189 (30.72)			
Elevated depressive symptoms	704 (21.35)	1332 (34.37)	148.59	1	< 0.001
**Body mass index**					
Baseline^a^	27.89 (4.27)	27.97 (5.41)	− 0.74	6990	0.45
Wave 6	28.11 (4.49)	28.15 (5.60)	− 0.23	4331	0.82
Wave 8	27.88 (4.44)	27.76 (5.61)	0.65	3212	0.51

^a^Combination of BMI measures collected at either wave 2 (for participants where blood was collected for genotyping at wave 2 (77%) and wave 4 (for participants where blood was collected at wave 4 (23%)).

### Educational attainment and BMI-PGS in relation to BMI trajectories

**Figure 1:**
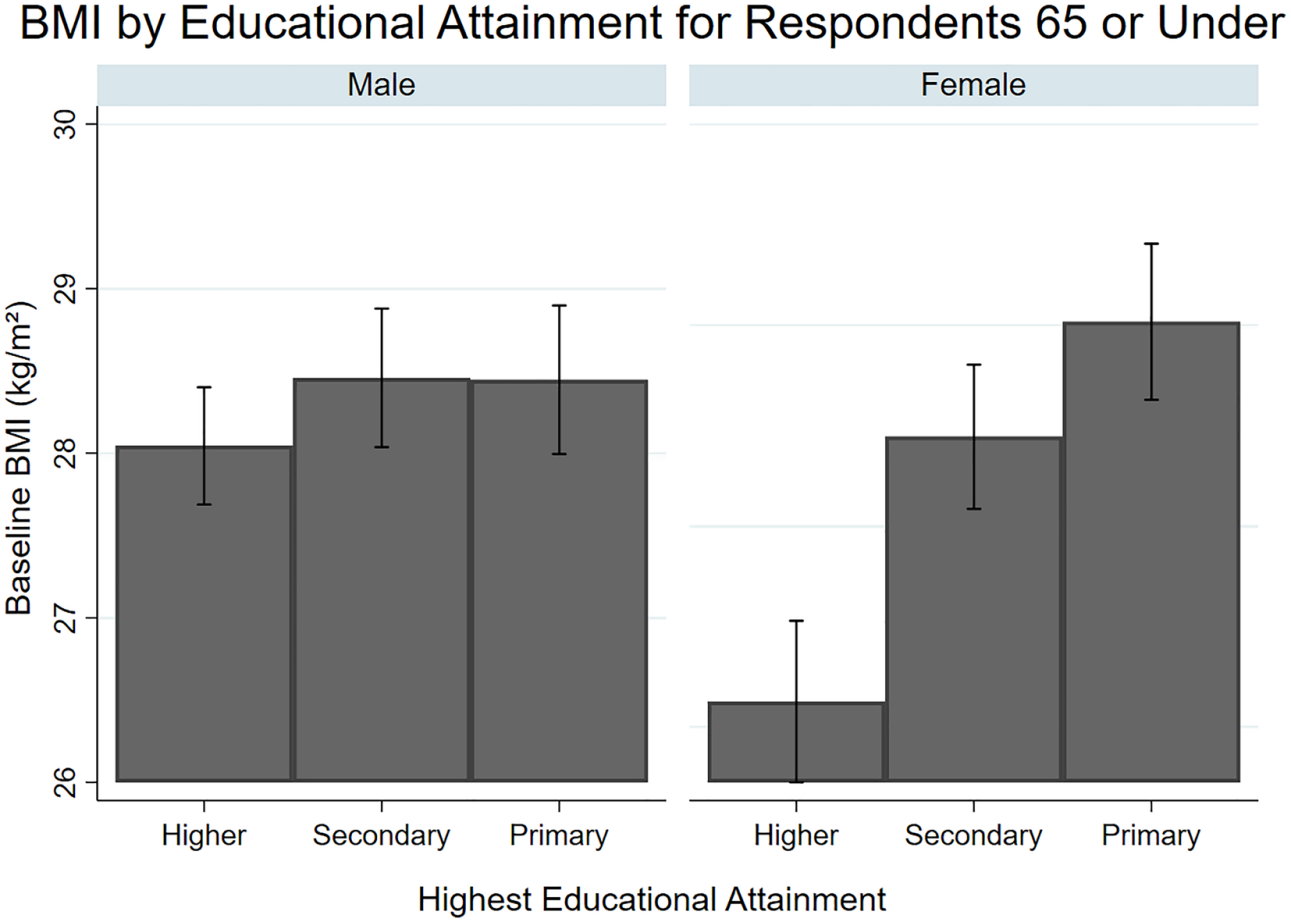
Mean Body Mass Index (BMI) at baseline for different levels of educational attainment between male and female respondents.

As compared to the group with a higher qualification, having a primary qualification was associated with higher BMI at baseline for women aged ≤ 65 years old (β = 1.25; 95%CI 0.64 –1.85) (Table 2), and women aged > 65 years old (β = 1.04; 95%CI 0.35 – 1.72) and men aged > 65 years old (β = 0.52; 95%CI 0.02 – 1.07) (Table 2). While having a secondary qualification was only associated with a higher BMI at baseline for women aged ≤ 65 years old (β = 1.02; 95%CI 0.45–1.60). Regarding interaction effects, a 1-SD increase in BMI-PGS was associated with a higher baseline BMI of 0.62 points in men aged > 65 of a secondary education as compared to those of a higher education (β = 0.62; 95% CI 0.00–1.24) (Fig. 1). For rate of change in BMI, in men aged ≤ 65 years, a secondary (β = 0.06; 95%CI 0.02 – 0.10) and primary qualification (β = 0.06; 95%CI 0.01 – 0.11) was associated with a steeper increase in BMI across the 12-year follow up than that found in the higher qualification group.

**Table 2:** Adjusted longitudinal mixed models exploring the main effect of polygenic score for BMI (BMI-PGS) and educational attainment, and interactions between these two variables in relation to BMI trajectories during the 12-year follow-up period.

	< 65 Years of age				> 65 Years of age			
	Men		Women		Men		Women	
	β	95% CI	β	95% CI	β	95% CI	β	95% CI
**Baseline**								
PGS	1.41***	1.10–1.71***	1.69***	1.29–2.10***	0.57**	0.25–0.88***	1.09***	0.59–1.59***
Higher degree	–	–	–	–	–	–	–	–
Secondary qualification	0.33	− 0.13 to 0.82	1.02***	0.45–1.60***	0.28	− 0.28 to -0.86	0.39	− 0.40 to 1.20
Primary qualification	0.42	− 0.08 to 0.93	1.25***	0.64–1.85***	0.52*	0.02–1.07*	1.04**	0.35–1.72**
PGS × higher qualification	–	–	–	–	–	–	–	–
PGS × secondary qualification	− 0.34	− 0.81 to 0.12	− 0.17	− 0.73 to 0.37	0.62*	0.09–1.16*	0.56	− 0.40 to 1.20
PGS × primary qualification	− 0.19	− 0.68 to 0.30	− 0.46	− 1.03 to 0.12	0.34	− 0.11 to 0.79	− 0.05	− 0.66 to 0.55
**Rate of change**								
PGS	− 0.00	− 0.03 to 0.02	− 0.01	− 0.02 to 0.05	0.01	− 0.04 to 0.05	0.04	− 0.02 to 0.10
Higher qualification	–	–	–	–	–	–	–	–
Secondary qualification	0.06**	0.02–0.10**	0.02	− 0.02 to 0.07	0.07	0.00–0.14	0.01	− 0.07 to 0.10
Primary qualification	0.06**	0.01–0.11**	0.05	− 0.00 to 0.10	0.02	− 0.04 to 0.09	− 0.07	− 0.14 to 0.01
PGS × higher qualification	–	–	–	–	–	–	–	–
PGS × secondary qualification	0.01	− 0.02 to 0.05	− 0.03	− 0.08 to 0.01	0.02	− 0.04 to 0.08	− 0.03	− 0.12 t o 0.04
PGS × primary qualification	0.01	− 0.03 to 0.06	0.01	− 0.04 to 0.06	− 0.03	− 0.09 to 0.03	− 0.01	− 0.08 to 0.06
**Variance**^a^								
Within-person	0.04	0.03–0.05	0.05	0.03–0.06	0.03	0.02–0.07	0.04	0.02–0.09
In initial status	15.89	14.74–17.13	24.44	22.78–26.22	12.79	11.22–14.58	20.72	18.58–23.12
In rate of change	0.03	− 0.06 to 0.11	0.01	− 0.11 to 0.11	− 0.06	− 0.26 to 0.14	0.15	− 0.04 to 0.33

The adjusted models were adjusted for 4 principal components to account for any ancestry differences in genetic structures that could bias the results, as well as; marital status, physical activity level, presence of longstanding limiting illness, self-reported health, depressive symptoms, and smoking status.

CI, confidence intervals; PGS, polygenic score; BMI, body mass index.

^a^The within-person variance is the overall residual variance in cognition that is not explained by the model. The initial status variance component is the variance of individuals’ intercepts about the intercept of the average person. The rate of change variance component is the variance of individual slopes about the slope of the average person.

×Represents an interaction between the two factors; interactions are presented based on multiplicative interaction model.

***p ≤ 0.001, **p ≤ 0.01, *p ≤ 0.05.

### Subjective social status (SSS) and BMI-PGS in relation to BMI trajectories

**Figure 2:**
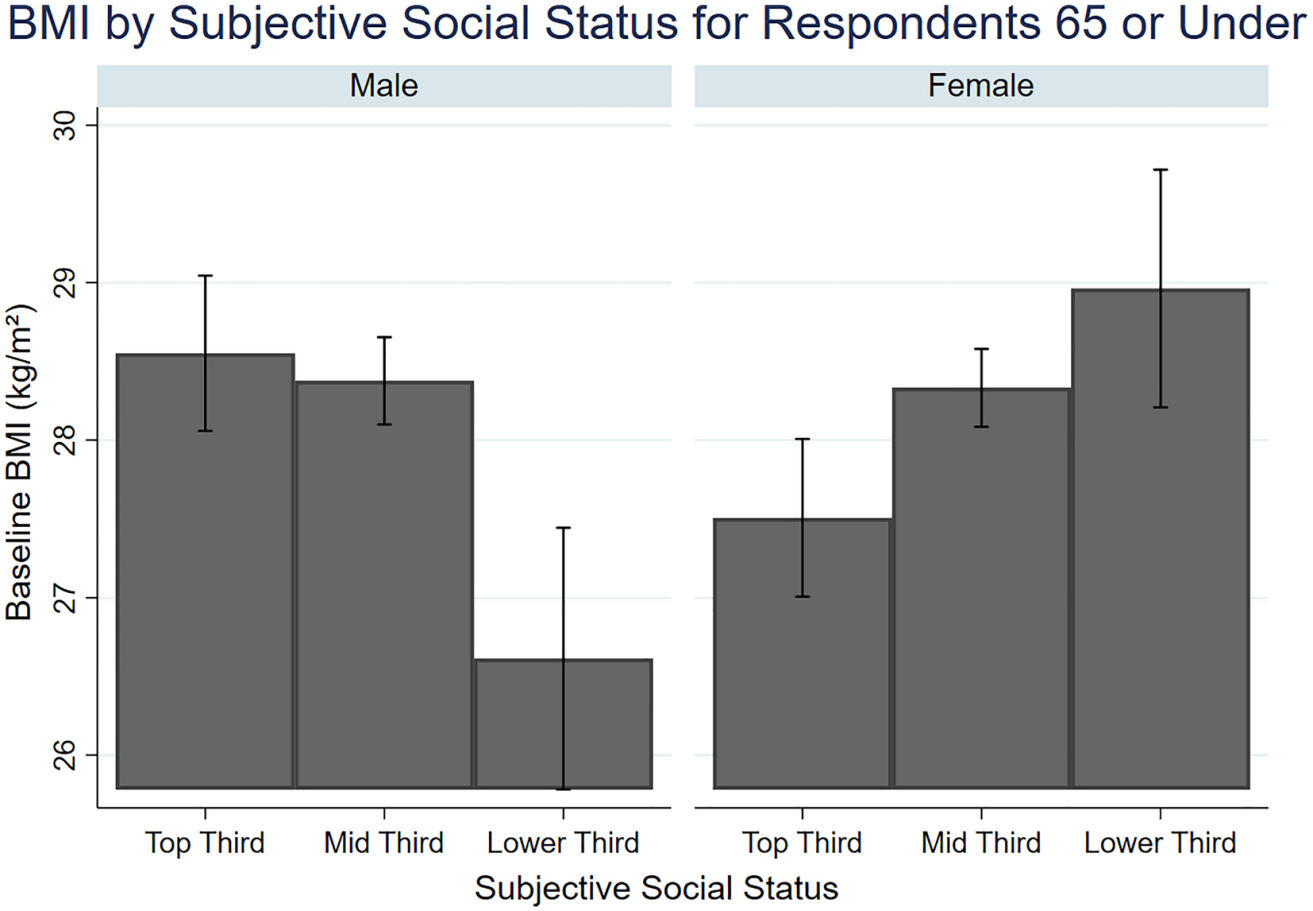
Mean Body Mass Index (BMI) at baseline for different levels of subjective social status between male and female respondents.

As compared to the highest SSS tertile, being in the middle tertile of SSS (β = 0.75; 95%CI 0.20–1.29) and bottom tertile of SSS (β = 1.16; 95%CI 0.08 – 2.24) was associated with a higher BMI at baseline for women aged ≤ 65 years old. In contrast, for men aged ≤ 65 years old, being in the bottom tertile of SSS was associated with a lower BMI at baseline (β = − 1.68; 95%CI − 2.55 to − 0.82) (Fig. 2). In both men and women aged > 65 years old, there was no association between SSS and baseline BMI (Table 3). There was an interaction effect between BMI-PGS and SSS on baseline BMI for women aged ≤ 65 years old, such that a 1-SD increase in BMI-PGS was associated with lower baseline BMI of 1.41 points for women in the bottom tertile of SSS (β = − 1.41; 95%CI − 2.46 to − 0.36) (Table 3). There were two interaction effects found for change in BMI over time. A higher BMI-PGS was associated with a reduction in BMI across time for men (aged ≤ 65) in the bottom tertile of SSS as compared to the highest SSS tertile (β = − 0.09; 95%CI − 0.17 to − 0.01) (Table 3). While for women aged > 65 years old, the BMI-PGS was associated with reductions in BMI for those in the bottom tertile of SSS (β = − 0.16; 95%CI − 0.32 − 0.01).

**Table 3:** Adjusted longitudinal mixed models exploring the main effect of polygenic score for BMI (BMI-PGS) and subjective social status, and interactions between these two variables in relation to BMI trajectories during the 12-year follow-up period.

	< 65 Years of age				> 65 Years of age			
	Men		Women		Men		Women	
	β	95% CI	β	95% CI	β	95% CI	β	95% CI
**Baseline**								
PGS	1.28***	0.87–1.68***	1.54***	1.01–2.01***	0.52*	0.10–0.94*	1.23***	0.61–1.84***
Top tertile	–	–	–	–	–	–	–	–
Middle tertile	− 0.33	− 0.81 to 0.15	0.75*	0.20–1.29*	0.05	− 0.49 to 0.60	0.37	− 0.35 to 1.09
Bottom tertile	− 1.68***	− **2.55 to **− 0.82***	1.16*	0.08–2.24*	0.44	− 0.45 to 1.35	0.37	− 0.74 to 1.47
PGS × top tertile	–	–	–	–	–	–	–	–
PGS × middle tertile	− 0.02	− 0.49 to 0.45	0.01	− 0.45 to 0.62	0.42	− 0.06 to 0.92	− 0.06	− 0.75 to 0.62
PGS × bottom tertile	0.41	− 0.44 to 1.26	− 1.41**	− **2.46 to **− 0.36**	0.64	− 0.45 to 1.41	0.20	− 0.91 to 1.31
**Rate of change**								
PGS	0.03	− 0.01 to 0.07	0.01	− 0.03 to 0.05	0.03	− 0.00 to 0.08	0.08	− 0.02 to .18
Top tertile	–	–	–	–	–	–	–	–
Middle tertile	0.03	− 0.02 to 0.08	− 0.02	− 0.07 to 0.03	− 0.03	− 0.09 to 0.03	0.01	− 0.09 to 0.11
Bottom tertile	0.02	− 0.08–0.11	0.03	− 0.06–0.12	− 0.01	− 0.14–0.12	− 0.08	− 0.24–0.07
PGS × top tertile	–	–	–	–	–	–	–	–
PGS × middle tertile	− 0.03	− 0.07 to 0.01	0.00	− 0.04 to 0.05	− 0.04	− 0.08 to 0.01	− 0.06	− 0.17 to − 0.04
PGS × bottom tertile	− 0.09*	− **0.17 to **− **0.01**	− 0.01	− 0.12 to 0.10	− 0.01	− 0.10 to 0.09	− 0.16*	− **0.33 to **− 0.07*
**Variance**^**a**^								
Within-person	0.05	0.03–0.06	0.05	0.03–0.06	0.03	0.02–0.07	0.04	0.03–0.09
In initial status	15.80	14.27 to 17.50	25.11	22.92–27.49	12.93	11.34–14.73	21.14	19.05–23.45
In rate of change	0.05	− 0.10 to 0.20	− 0.03	− 0.18 to 0.12	− 0.06	− 0.26 to 0.13	0.13	− 0.06 to 0.32

The adjusted models were adjusted for 4 principal components to account for any ancestry differences in genetic structures that could bias the results, as well as; marital status, physical activity level, presence of longstanding limiting illness, self-reported health, depressive symptoms, and smoking status. Adjusted models used robust standard errors to relax the assumption that standard errors carried identical and equal distributions, due to the presence of heteroscedascity.

CI, confidence intervals; PGS, polygenic score; BMI, body mass index.

^a^The within-person variance is the overall residual variance in cognition that is not explained by the model. The initial status variance component is the variance of individuals’ intercepts about the intercept of the average person. The rate of change variance component is the variance of individual slopes about the slope of the average person.

×Represents an interaction between the two factors; interactions are presented based on multiplicative interaction model.

***p ≤ 0.001, **p ≤ 0.01, *p ≤ 0.05.

### Income and BMI-PGS in relation to BMI trajectories

Compared with the highest income tertile, women who were aged > 65 years old in the intermediate (β = 0.81, 95%CI 0.09–1.53) and lowest income tertiles groups (β = 0.86, 95%CI 0.18 - − 1.53) had higher baseline BMI values (Table 4). While for men aged > 65 years old, the intermediate income tertile showed lower baseline BMI values (β = − 0.67, 95%CI − 1.21 - − 0.13). There was an interaction effect between BMI-PGS and income for BMI at baseline for men aged ≤ 65, such that a 1-SD increase in BMI-PGS was associated with a lower baseline BMI value of 0.72 points for men in the lowest tertile of income but not in the highest (β = − 0.72, 95%CI − 1.21 to − 0.23) (Table 4). There were no significant direct effects or interaction effects of income on the rate of change in BMI over the 12-year follow up for either men or women.

**Table 4:** Adjusted longitudinal mixed models exploring the main effect of polygenic score for BMI (BMI-PGS) and income, and interactions between these two variables in relation to BMI trajectories during the 12-year follow-up period.

	< 65 Years of age				> 65 Years of age			
	Men		Women		Men		Women	
	β	95% CI	β	95% CI	β	95% CI	β	95% CI
**Baseline**								
PGS	1.41***	1.12 to 1.69***	1.52***	1.17–1.85***	0.85***	0.47–1.22***	1.33***	0.75–1.91***
High income	–	–	–	–	–	–	–	–
Intermediate income	0.09	− 0.36 to 0.56	0.51	− 0.04 to 1.06	− 0.67*	− **1.21 to **− 0.13*	0.81*	0.09–1.53*
Low income	− 0.39	− 0.91 to 0.13	0.55	− 0.02 to 1.13	− 0.35	− 0.87 to 0.18	0.86*	0.18–1.48*
PGS × high income	–	–	–	–	–	–	–	–
PGS × intermediate income	0.17	− 0.29 to 0.63	0.14	− 0.41 to 0.69	− 0.09	− 0.61 to 0.43	− 0.24	− 1.01 to 0.51
PGS × Low income	− 0.72**	− **1.21 to **− 0.23**	− 0.22	− 0.78 to 0.33	0.22	− 0.28 to 0.73	− 0.17	− 0.84 to 0.50
**Rate of change**								
PGS	− 0.00	− 0.02 to 0.02	0.01	− 0.01 to 0.04	0.01	− 0.02 to 0.05	− 0.02	− 0.09 to 0.06
High income	–	–	–	–	–	–	–	–
Intermediate income	0.03	− 0.01 to 0.08	0.03	− 0.02 to 0.07	0.05	− 0.01 to 0.12	− 0.03	− 0.08 to 0.08
Low income	0.03	− 0.02 to 0.08	0.03	− 0.01 to .08	0.06	− 0.00 to 0.12	− 0.04	− 0.12 to 0.04
PGS × high income	–	–	–	–	–	–	–	–
PGS × intermediate income	0.00	− 0.04 to 0.04	− 0.04	− 0.08 to 0.01	0.01	− 0.06 to 0.07	0.06	− 0.03 to 0.15
PGS × low income	0.01	− 0.03 to 0.06	0.02	− 0.02 to 0.08	− 0.03	− 0.09 to 0.03	0.04	− 0.05 to 0.13
**Variance**^**a**^								
Within-person	0.04	0.03–0.05	0.05	0.04–0.06	0.03	0.02–0.07	0.04	0.02–0.08
In initial status	16.02	14.88–17.25	25.22	23.57–26.99	13.59	11.33–14.67	20.95	18.95–23.16
In rate of change	0.06	− 0.02 to .14	− 0.04	− 0.15 to 0.07	0.07	− 0.02 to 0.15	− 0.02	− 0.12 to 0.08

The adjusted models were adjusted for 4 principal components to account for any ancestry differences in genetic structures that could bias the results, as well as; marital status, physical activity level, presence of longstanding limiting illness, self-reported health, depressive symptoms, and smoking status.

CI, confidence intervals; PGS, polygenic score; BMI, body mass index.

^a^The within-person variance is the overall residual variance in cognition that is not explained by the model. The initial status variance component is the variance of individuals’ intercepts about the intercept of the average person. The rate of change variance component is the variance of individual slopes about the slope of the average person.

×Represents an interaction between the two factors; interactions are presented based on multiplicative interaction model.

***p ≤ 0.001, **p ≤ 0.01, *p ≤ 0.05.

### Sensitivity analyses

After mutiple imputation for missing data, we observed that as compared to a higher qualification, men with a primary level education had a higher BMI at baseline in both the > 65 age group (β = 0.51; 95%CI 0.08–1.11) and ≤ 65 age group (β = 0.49; 95%CI 0.00–0.98) (Supplementary Table 3). For SSS, the interaction between BMI-PGS and the bottom tertile of SSS on rate of change in BMI in men aged > 65 uncovered in the main analyses was attenuated towards null. Moreover, women aged ≤ 65 and in the lowest tertile of SSS, no longer showed higher BMI at baseline (Supplementary Table 4). For income, women aged ≤ 65 and in the mid and lower income tertiles did not show higher BMI at baseline as in the main analyses (Supplementary Table 5).

## Discussion

To our knowledge, the present study is the first to investigate GxE interactions between an aggregate measure of genetic risk for BMI and three dimensions of socioeconomic position the rate of change in BMI across older adulthood. Consistent with previous findings^15,45^, our results showed that a higher BMI-PGS was associated with higher baseline BMI (~ 64 years of age) in both men and women. However, contrary to our second hypothesis, BMI-PGS was not significantly associated with a higher rate of change in BMI during the 12-year follow-up period. These results may imply that polygenic factors that contribute to BMI variation in mid-to-older adulthood may differ from those which influence BMI fluctuations at older ages.

Consistent with previous findings, our results demonstrate that lower levels of educational attainment, SSS and income were associated with higher baseline BMI more so in women than in men^46^. Moreover, males with lower SSS and incomes even showed lower BMI values at baseline. Hence, women in mid-to-late adulthood may be more exposed to the limited opportunities for physical activity and lower quality diet present in lower socio-economic settings than are men, and therefore show a stronger social gradient in BMI outcomes than men of the same social standing^47,48^. For instance, the well reported ‘gender pension gap’ or the tendency for women in the UK to enter retirement with a lower private pension wealth and income retirement than men across the social gradient^49^, may result in greater obesogenic exposures for women in later adulthood. With fewer resources and larger occupational demands in later life, women may be exposed to a greater risk of adiposity development across older adulthood than men of a similar social position^50^. Nonetheless, a reverse causal effect between obesity and labour market outcomes may be present, as findings have consistently shown that developing obesity influences a women’s labour market outcomes, and hence income and SSS, to a greater extent than men^51,52^. A novel finding was also that SSS was only associated with baseline BMI in adults aged 65 or less. It has been proposed that those who perceive themselves to have fewer social and economic lower resources may be more exposed and more susceptible to the obesogenic environment^20^. Hence, as younger populations have developed in the context of a more obesogenic environment, the influence of self-perceived resources might therefore be stronger in younger age groups^20^. This finding highlights how BMI inequalities may vary across specific age ranges, and future investigations may benefit from exploring social gradients across both gender, SEP measures, and varying stages of the life course.

Three GxE interactions between socioeconomic positioning and BMI-PGS were observed. First, the BMI-PGS showed a stronger association with baseline BMI in men (aged ≤ 65) with a secondary qualification than those with a higher qualification. Hence, a lower educational attainment may accentuate genetic risk for BMI as less education may place individuals within more obesogenic environments where opportunities to express underlying genetic risk are more pervasive^17,21^. Similarly, we further observed that in men of a lower income (aged ≤ 65) a higher BMI-PGS scores were associated with higher baseline BMI values, as compared to those in a higher income group. Finally, for women aged ≤ 65 or younger, a higher BMI-PGS was associated with a lower baseline BMI only for those in the lowest SSS tertile. Together, these findings might suggest that women’s expression of polygenic risk towards a higher BMI is more influenced by subjective measures of social standing than tangible levels of education or income. Nonetheless, while these findings provide evidence that the expression of polygenic predisposition may be sensitive to the socioeconomic environment, it is noteworthy that, similar Tyrell et al^21^, the present GxE interactions produced smaller effect sizes than the direct effects of socioeconomic status on BMI.

### Strengths and limitations

In the present study, we analysed a large population-based cohort who are representative of older adults in England. Confidence in these findings is also strengthened by using LMMs, which are an optimal way to describe the changes in continuous dependent variables over time taking into account intra and inter-individual variation. Moreover, the sample utilised in the present study was appropriate for evaluating the stated hypotheses as it was substantially larger or similar in size to samples used in previous work^19,20^.

Nonetheless, given the observational nature of this study, we cannot infer causality or eliminate the role of residual confounding. It is feasible that PGS utilised in the present study, having encompassed hundreds to thousands of common variants, may have accumulated noise which masks the true associations with changes in BMI over time^16^. Moreover, the poor generalizability of genetic studies across populations is noteworthy as PGSs are predominately based on in European participants^40^. Moreover, as GWASs do not, by design, capture other structural variants beyond SNPs such as rare variants, poorly tagged or multiple independent variants, G × G interaction, epigenetics and gene-environment correlation^53^. Moreover, to avoid overfitting the present GxE models we also were unable to adjust our analyses for interactions between the covariates and the present BMI-PGS and SEP variables, as advised by Keller et al.^53^ Finally, the use of height data from Wave 6 (2012) to calculate BMI at wave 8 (2016) may have affected the validity of the final follow-up BMI measures.

## Conclusion

The BMI-PGS was associated with higher BMI at baseline (~64 years old) but not with the rate of change in BMI over the 12-year follow-up period. Moreover, women’s BMI appeared to be more adversely affected by lower education, lower SSS, and less income than men’s BMI. Crucially, the current results highlight the potential for educational attainment, SSS, and income to influence BMI in adulthood through interaction with a BMI-PGS, although effect sizes were small. Taken together, lower socioeconomic positioning may adversely influence BMI in adulthood both independently and through accentuation of genetic risk. However, further research must clarify the extent to which cumulative measures of socioeconomic conditions may influence the expression of genetic propensity towards a higher BMI.

## Supplementary Information

Supplementary Information.

## Data Availability

The ELSA data are available in public, open-access repository (the UK Data Archive) which is freely available and can be accessed at https://discover.ukdataservice.ac.uk.
