# Supplementary material for: Genetic propensity for obesity, socioeconomic position, and trajectories of body mass index in older adults
Source: Sci Rep. 2021 Oct 13;11:20276. doi: 10.1038/s41598-021-99332-7 (PMC8514538; doi:10.1038/s41598-021-99332-7)
Supplement: Supplementary file 1 — Supplementary Information. [file 41598_2021_99332_MOESM1_ESM.docx]

**Supplementary Table 1. Descriptive of missing data for male and female respondents with complete polygenic risk scores**

| Variable | Missing (N) | Missing (%) | Complete (N) | Complete (%) |
| --- | --- | --- | --- | --- |
|  |  |  |  |  |
| *Social Position Variables* |  |  |  |  |
| Income | 215 | 2.99 | 6968 | 97.01 |
| Educational Attainment | 624 | 8.69 | 6559 | 91.31 |
| Subjective Social Status | 369 | 5.14 | 6814 | 94.86 |
| *Covariate Variables* |  |  |  |  |
| Presence of Illness | 0 | 0.00 | 7183 | 100.00 |
| Marital Status | 0 | 0.00 | 7183 | 100.00 |
| Smoking Status | 29 | 0.40 | 7154 | 99.60 |
| Self-Reported Health | 1 | 0.01 | 7182 | 99.99 |
| Physical Activity | 15 | 0.21 | 7168 | 99.79 |
| Depressive Symptoms | 9 | 0.13 | 7174 | 99.87 |
| *Body Mass Index* |  |  |  |  |
| Baseline^a^ | 261 | 3.60 | 6922 | 96.40 |
| Wave 6 | 2850 | 39.68 | 4333 | 60.32 |
| Wave 8 | 3969 | 55.25 | 3214 | 44.74 |

^a^ Combination of BMI measures collected at either wave 2 (for participants where blood was collected for genotyping at wave 2 (77%) and wave 4 (for participants where blood was collected at wave 4 (23%))

**Supplementary Table 2. Imputed sample characteristics of ELSA participants**

|  | | N(%) / Mean (SD) | N(%) / Mean (SD) |
| --- | --- | --- | --- |
| Sample Characteristics | | Men (n=3304) | Women (n=3879) |
| Current Smoker | | 515 (15.59) | 656 (16.91) |
|  | |  |  |
| Not married | | 780 (23.61) | 1466 (37.79) |
|  | |  |  |
| Income | |  |  |
|  | Highest Tertile | 1285 (38.89) | 1273 (32.82) |
|  | Middle Tertile | 1013 (30.66) | 1157 (29.83) |
|  | Lowest Tertile | 1006 (30.45) | 1449 (37.35) |
|  |  |  |  |
| Highest Educational Attainment | |  |  |
|  | Higher Qualification | 1170 (35.41) | 943 (24.31) |
|  | Secondary Qualification | 852 (25.79) | 1198 (30.88) |
|  | Primary Qualification | 1282 (38.80) | 1738 (44.81) |
|  |  |  |  |
| Subjective Social Status | |  |  |
|  | Top Tertile | 654 (19.79) | 606 (15.62) |
|  | Middle Tertile | 2321 (70.25) | 2896 (74.66) |
|  | Lower Tertile | 329 (9.96) | 377 (9.72) |
|  |  |  |  |
| Longstanding Illness present | | 1758 (74.88) | 2177 (56.17) |
|  |  |  |  |
| Poor Self-Reported Health | | 772 (23.37) | 938 (24.18) |
|  |  |  |  |
| Physical Activity | |  |  |
|  | Sedentary or Light Activity | 533 (16.13) | 804 (20.73) |
|  | Moderate Activity | 1579 (47.79) | 1882 (48.52) |
|  | Vigorous Activity | 1192 (36.08) | 1193 (30.76) |
|  |  |  |  |
| Elevated Depressive Symptoms | | 706 (21.37) | 1333 (34.36) |
|  |  |  |  |
| Body Mass Index (BMI) | |  |  |
|  | Baseline^a^ | 27.81 (4.09) | 27.91 (5.22) |
|  | Wave 6 | 28.042 (4.40) | 28.16 (5.58) |
|  | Wave 8 | 30.13 (11.39) | 29.84 (11.73) |

^a^ Combination of BMI measures collected at either wave 2 (for participants where blood was collected for genotyping at wave 2 (77%) and wave 4 (for participants where blood was collected at wave 4 (23%))

**Supplementary Table 3. Unadjusted longitudinal mixed models exploring the main effect of polygenic score for BMI and educational attainment, and interaction between these two variables, in relation to BMI trajectories during the 12-year follow-up period**

|  |  | **<65 Years Old** | | | | **>65 Years Old** | | | | | |
| --- | --- | --- | --- | --- | --- | --- | --- | --- | --- | --- | --- |
|  |  | **Men** | | **Women** | | **Men** | | | **Women** | |  |
|  |  | **β** | **95% CI** | **β** | **95% CI** | **β** | **95% CI** | | **β** | **95% CI** |  |
| *Baseline* | |  |  |  |  |  | |  |  |  |  |
|  | PGS | **1.40***** | **1.11- 1.69** | **1.51***** | **1.16-1.89** | **0.87***** | **0.46-1.29** | | **1.40***** | **1.11- 1.98** |  |
|  | Higher degree | - | - |  |  | - | - | | - | - |  |
|  | Secondary qualification | 0.23 | -0.23-0.70 | **0 .80**** | **0.24-1.36** | **-0.58**** | **-1.14--0.02** | | **0.82*** | **0.09-1.56** |  |
|  | Primary qualification | -0.11 | -0.62-0.39 | **1.01***** | **0.44- 1.58** | -0.33 | -0.87-0.21 | | **0.83*** | **0.15-1.50** |  |
|  | |  |  |  |  |  | |  |  |  |  |
|  | PGS × Higher degree | - | - | - | - | - | - | | - | - |  |
|  | PGS × Secondary qualification | 0.19 | -0.27-0.67 | 0.31 | -0.25-0.88 | -0.12 | -0.67-0.43 | | -0.30 | -0.27-0.67 |  |
|  | PGS × Primary qualification | **-0.65**** | **-1.14- -0.15** | -0.08 | -0.66-0.50 | 0.19 | -0.34-0.74 | | -0.17 | -0.86-0.51 |  |
|  |  |  |  |  |  |  |  | |  |  |  |
| *Rate of change* | |  |  |  |  |  | |  |  |  |  |
|  | PGS | -0.00 | -0.02-0.02 | 0.01 | -0.01-0.04 | 0.01 | -0.03-0.05 | | -.02 | 0.09-0.05 |  |
|  | Higher degree | - | - | - | - | - | - | | - | - |  |
|  | Secondary qualification | 0.03 | - 0.01-0.08 | 0.03 | -0.01-0.07 | 0.05 | -0.01-0.12 | | -0.00 | -0.08-0.07 |  |
|  | Primary qualification | 0.02 | -0.02-0.06 | 0.03 | -0.01-.08 | 0.06 | -0.01-0.12 | | -0.04 | -0.11-0.04 |  |
|  |  |  |  |  |  |  |  | |  |  |  |
|  | PGS × Higher Degree | - | - |  |  | - | - | | - | - |  |
|  | PGS × Secondary | 0.00 | -0.04-0.04 | -0.03 | -0.08-0.01 | 0.00 | -0.06-0.07 | | 0.06 | -0.02- 0.15 |  |
|  | PGS × Primary Qualification | 0.01 | -0.03-0.06 | 0.02 | -0.03-0.06 | -0.03 | -0.09-0.03 | | 0.04 | -0.04-0.13 |  |
|  |  |  |  |  |  |  |  | |  |  |  |
| *Variance ^a^* | | *Variance ^a^* |  |  |  |  | |  |  |  |  |
|  | Within-person | 0.04 | 0.03-0.05 | 0.05 | 0.04-0.06 | 0.03 | 0.02-0.05 | | 0.04 | 0.03-0.07 |  |
|  | In initial status | 16.71 | 15.53-17.98 | 27.10 | 25.34-28.97 | 13.59 | 12.39-14.85 | | 22.13 | 20.35-24.06 |  |
|  | In rate of change | 0.06 | -0.02-.14 | -0.04 | -0.15-0.07 | -0.07 | -0.17-0.04 | | 0.09 | -0.07-0.26 |  |

CI, confidence intervals; PGS, polygenic score; BMI, body mass index

The adjusted models were adjusted for 4 principal components to account for any ancestry differences in genetic structures that could bias the results, as well as; marital status, physical activity level, presence of longstanding limiting illness, self-reported health, depressive symptoms, and smoking status.

^a^ The within-person variance is the overall residual variance in cognition that is not explained by the model. The initial status variance component is the variance of individuals’ intercepts about the intercept of the average person. The rate of change variance component is the variance of individual slopes about the slope of the average person.

× represents an interaction between the two factors; interactions are presented based on multiplicative interaction model

****p*≤0.001, ***p*≤0.01, **p*≤0.05

**Supplementary Table 4. Unadjusted longitudinal mixed models exploring the main effect of polygenic score for BMI and subjective social status, and interaction between these two variables, in relation to BMI trajectories during the 12-year follow-up period**

|  |  | **<65 Years of Age** | | | | | | **>65 Years of Age** | | | | | |
| --- | --- | --- | --- | --- | --- | --- | --- | --- | --- | --- | --- | --- | --- |
|  |  | **Men** | | | **Women** | | | **Men** | | | **Women** | |  |
|  |  | **β** | **95% CI** | | **β** | **95% CI** | | **β** | **95% CI** | | **β** | **95% CI** |  |
| *Baseline* | |  | |  |  | |  |  | |  |  |  |  |
|  | PGS | **1.28***** | **0.83-1.72** | | **1.52***** | **0.97-2.06** | | **0.48*** | **-0.00-.97** | | **1.25***** | **0.52-1.99** |  |
|  | Top tertile | - | - | | - | - | | - | - | | - | - |  |
|  | Middle tertile | -0.20 | -0.69-0.28 | | **0.94**** | **0.33-1.55** | | 0.11 | -0.46-0.68 | | 0.44 | -0.34-1.22 |  |
|  | Bottom tertile | **-1.04**** | **-1.85--0.21** | | **2.12***** | **1.13-3.11** | | 0.61 | -0.24-1.47 | | 0.56 | -0.59-1.73 |  |
|  | |  | |  |  | |  |  | |  |  |  |  |
|  | PGS × Top tertile | - | - | | - | - | | - | - | | - | - |  |
|  | PGS × Middle tertile | 0.03 | -0.47-0.53 | | 0.20 | -0.40-0.81 | | 0.48 | -0.06-1.04 | | -0.04 | -0.83-0.74 |  |
|  | PGS × Bottom tertile | 0.48 | -0.35-1.33 | | **-1.22*** | **-2.22--0.23** | | 0.56 | -0.27-1.40 | | 0.27 | -0.92-1.47 |  |
|  |  |  |  | |  |  | |  |  | |  |  |  |
| *Rate of change* | |  | |  |  | |  |  | |  |  |  |  |
|  | PGS | 0.02 | -0.01-0.07 | | 0.01 | -0.03-0.05 | | 0.03 | -.02-0.09 | | **0.08*** | **0.00-0.17** |  |
|  | Top tertile | - | - | | - | - | | - | - | | - | - |  |
|  | Middle tertile | 0.03 | -0.01-0.08 | | -0.01 | -0.06-0.03 | | -0.02 | -0.09-0.04 | | 0.00 | -0.08-0.09 |  |
|  | Bottom tertile | 0.02 | -0.06-0.10 | | 0.03 | -0.04-0.12 | | -0.01 | -0.11-0.10 | | -0.08 | -0.22-0.05 |  |
|  |  |  |  | |  |  | |  |  | |  |  |  |
|  | PGS × Top tertile | - | - | | - | - | | - | - | | - | - |  |
|  | PGS × Middle tertile | -0.03 | -0.07-0.01 | | 0.00 | -0.04-0.05 | | -0.03 | -0.10-0.02 | | -0.06 | -0.15-0.03 |  |
|  | PGS × Bottom tertile | **-0.09*** | **-0.17- -0.001** | | -0.00 | -0.09-0.08 | | -0.00 | -0.09-0.09 | | **-0.16*** | **-0.32- -0.01** |  |
|  |  |  |  | |  |  | |  |  | |  |  |  |
| *Variance ^a^* | |  | |  |  | |  |  | |  |  |  |  |
|  | Within-person | 0.04 | 0.03-0.06 | | 0.05 | 0.03- 0.06 | | 0.03 | 0.02-0.04 | | 0.04 | 0.02-0.07 |  |
|  | In initial status | 16.58 | 15.41. 17.84 | | 27.10 | 25.36-28.96 | | 13.56 | 12.37-14.86 | | 22.36 | 20.52-24.37 |  |
|  | In rate of change | 0.05 | -0.04-0.13 | | -0.05 | -0.16-0.06 | | -0.07 | -0.18-0.04 | | 0.06 | -0.10-0.23 |  |

CI, confidence intervals; PGS, polygenic score; BMI, body mass index

The adjusted models were adjusted for 4 principal components to account for any ancestry differences in genetic structures that could bias the results, as well as; marital status, physical activity level, presence of longstanding limiting illness, self-reported health, depressive symptoms, and smoking status. Adjusted models used robust standard errors to relax the assumption that standard errors carried identical and equal distributions, due to the presence of heteroscedascity.

^a^ The within-person variance is the overall residual variance in cognition that is not explained by the model. The initial status variance component is the variance of individuals’ intercepts about the intercept of the average person. The rate of change variance component is the variance of individual slopes about the slope of the average person.

× represents an interaction between the two factors; interactions are presented based on multiplicative interaction model

****p*≤0.001, ***p*≤0.01, **p*≤0.05

**Supplementary Table 5. Adjusted longitudinal mixed models exploring the main effect of polygenic score for BMI and income, and interaction between these two variables, in relation to BMI trajectories during the 12-year follow-up period**

|  |  | **<65 Years of Age** | | | | **>65 Years of Age** | | | | | |
| --- | --- | --- | --- | --- | --- | --- | --- | --- | --- | --- | --- |
|  |  | **Men** | | **Women** | | **Men** | | | **Women** | |  |
|  |  | **β** | **95% CI** | **β** | **95% CI** | **β** | **95% CI** | | **β** | **95% CI** |  |
| *Baseline* | |  |  |  |  |  | |  |  |  |  |
|  | PGS | **1.40***** | **1.11- 1.69** | **1.51***** | **1.16-1.89** | **0.87***** | **0.46-1.29** | | **1.40***** | **1.11- 1.98** |  |
|  | High income | - | - |  |  | - | - | | - | - |  |
|  | Intermediate income | 0.23 | -0.23-0.70 | **0 .80**** | **0.24-1.36** | **-0.58**** | **-1.14--0.02** | | **0.82*** | **0.09-1.56** |  |
|  | Low income | -0.11 | -0.62-0.39 | **1.01***** | **0.44- 1.58** | -0.33 | -0.87-0.21 | | **0.83*** | **0.15-1.50** |  |
|  | |  |  |  |  |  | |  |  |  |  |
|  | PGS × High income | - | - | - | - | - | - | | - | - |  |
|  | PGS × Intermediate income | 0.19 | -0.27-0.67 | 0.31 | -0.25-0.88 | -0.12 | -0.67-0.43 | | -0.30 | -0.27-0.67 |  |
|  | PGS × Low income | **-0.65**** | **-1.14- -0.15** | -0.08 | -0.66-0.50 | 0.19 | -0.34-0.74 | | -0.17 | -0.86-0.51 |  |
|  |  |  |  |  |  |  |  | |  |  |  |
| *Rate of change* | |  |  |  |  |  | |  |  |  |  |
|  | PGS | -0.00 | -0.02-0.02 | 0.01 | -0.01-0.04 | 0.01 | -0.03-0.05 | | -.02 | 0.09-0.05 |  |
|  | High income | - | - | - | - | - | - | | - | - |  |
|  | Intermediate income | 0.03 | - 0.01-0.08 | 0.03 | -0.01-0.07 | 0.05 | -0.01-0.12 | | -0.00 | -0.08-0.07 |  |
|  | Low income | 0.02 | -0.02-0.06 | 0.03 | -0.01-.08 | 0.06 | -0.01-0.12 | | -0.04 | -0.11-0.04 |  |
|  |  |  |  |  |  |  |  | |  |  |  |
|  | PGS × High income | - | - |  |  | - | - | | - | - |  |
|  | PGS × Intermediate income | 0.00 | -0.04-0.04 | -0.03 | -0.08-0.01 | 0.00 | -0.06-0.07 | | 0.06 | -0.02- 0.15 |  |
|  | PGS × Low income | 0.01 | -0.03-0.06 | 0.02 | -0.03-0.06 | -0.03 | -0.09-0.03 | | 0.04 | -0.04-0.13 |  |
|  |  |  |  |  |  |  |  | |  |  |  |
| *Variance ^a^* | |  |  |  |  |  | |  |  |  |  |
|  | Within-person | 0.04 | 0.03-0.05 | 0.05 | 0.04-0.06 | 0.03 | 0.02-0.05 | | 0.04 | 0.03-0.07 |  |
|  | In initial status | 16.71 | 15.53-17.98 | 27.10 | 25.34-28.97 | 13.59 | 12.39-14.85 | | 22.13 | 20.35-24.06 |  |
|  | In rate of change | 0.06 | -0.02-.14 | -0.04 | -0.15-0.07 | -0.07 | -0.17-0.04 | | 0.09 | -0.07-0.26 |  |

CI, confidence intervals; PGS, polygenic score; BMI, body mass index

The adjusted models were adjusted for 4 principal components to account for any ancestry differences in genetic structures that could bias the results, as well as; marital status, physical activity level, presence of longstanding limiting illness, self-reported health, depressive symptoms, and smoking status.

^a^ The within-person variance is the overall residual variance in cognition that is not explained by the model. The initial status variance component is the variance of individuals’ intercepts about the intercept of the average person. The rate of change variance component is the variance of individual slopes about the slope of the average person.

× represents an interaction between the two factors; interactions are presented based on multiplicative interaction model

****p*≤0.001, ***p*≤0.01, **p*≤0.05

|  |  | **<65 Years of Age** | | | | **>65 Years of Age** | | | | | |
| --- | --- | --- | --- | --- | --- | --- | --- | --- | --- | --- | --- |
|  |  | **Men** | | **Women** | | **Men** | | | **Women** | |  |
|  |  | **β** | **95% CI** | **β** | **95% CI** | **β** | **95% CI** | | **β** | **95% CI** |  |
| *Baseline* | |  |  |  |  |  | |  |  |  |  |
|  | PGS | **1.39***** | **1.08, 1.70** | **1.69***** | **1.29, 2.08** | **0.57**** | **0.18, 0.96** | | **0.99***** | **0.43, 1.55** |  |
|  | Higher Degree | - | - | - | - | - | - | | - | - |  |
|  | Secondary Qualification | 0.26 | -0.20, 0.72 | **.96***** | **0.42, 1.51** | 0.41 | -0.20, 1.01 | | 0.56 | -0.20, 1.34 |  |
|  | Primary Qualification | **0.49*** | **0.00, 0.98** | **1.22***** | **0.65, 1.79** | **0.51*** | **0.08, 1.11** | | **1.03**** | **0.35, 1.71** |  |
|  | |  |  |  |  |  | |  |  |  |  |
|  | PGS × Higher Degree | - | - | - | - | - | - | | - | - |  |
|  | PGS × Secondary Qualification | -0.26 | -0.73, 0.19 | -0.09 | -0.64, 0.45 | **0.61*** | **0.01, 1.21** | | 0.76 | -0.00, 1.53 |  |
|  | PGS × Primary Qualification | -0.06 | -0.53, 0.41 | -0.45 | -1.02, 0.10 | 0.32 | -0.17, 0.81 | | 0.07 | -0.63, 0.65 |  |
|  |  |  |  |  |  |  |  | |  |  |  |
| *Rate of change* | |  |  |  |  |  | |  |  |  |  |
|  | PGS | -0.01 | -0.04, 0.03 | 0.01 | -0.02, 0.05 | 0.01 | -0.03, 0.05 | | 0.04 | -0.02, 0.11 |  |
|  | Higher Degree | - | - | - | - | - | - | | - | - |  |
|  | Secondary Qualification | **0.06**** | **0.02, 0.11** | 0.01 | -0.03, 0.06 | 0.06 | -0.00, 0.13 | | -0.01 | -0.09, 0.07 |  |
|  | Primary Qualification | **0.06**** | **0.01, 0.11** | 0.06 | 0.01, 0.11 | 0.02 | -0.04, 0.08 | | -0.08 | -0.15, -0.01 |  |
|  |  |  |  |  |  |  |  | |  |  |  |
|  | PGS × Higher Degree | - | - | - | - | - | - | | - | - |  |
|  | PGS × Secondary Qualification | 0.02 | -0.02, 0.06 | -0.04 | -0.08, 0.00 | 0.02 | -0.04, 0.09 | | -0.03 | -0.12, 0.06 |  |
|  | PGS × Primary Qualification | -0.01 | -0.05, 0.05 | 0.01 | -0.04, 0.06 | -0.02 | -0.08, 0.04 | | -0.02 | -0.10, 0.08 |  |
|  |  |  |  |  |  |  |  | |  |  |  |
| *Variance ^a^* | |  |  |  |  |  | |  |  |  |  |
|  | Within-person | 0.04 | 0.04, 0.06 | 0.05 | 0.03, 0.06 | 0.03 | 0.02, 0.05 | | 0.04 | 0.02, 0.07 |  |
|  | In initial status | 15.93 | 14.81, 17.13 | 24.89 | 23.31, 26.59 | 12.78 | 11.68, 13.99 | | 20.83 | 19.15. 22.66 |  |
|  | In rate of change | 0.05 | -0.03, 0.13 | -0.03 | -0.13, 0.07 | -0.06 | -0.17, 0.04 | | 0.16 | -0.02, 0.32 |  |

**Supplementary Table 6. Longitudinal mixed models exploring the main effect of polygenic score for BMI and imputed educational attainment, and interaction between these two variables, in relation to BMI trajectories during the 12-year follow-up period adjusted for imputed covariates**

CI, confidence intervals; PGS, polygenic score

The adjusted models were adjusted for 4 principal components to account for any ancestry differences in genetic structures that could bias the results, as well as; marital status, physical activity level, presence of longstanding limiting illness, self-reported health, depressive symptoms, and smoking status.

^a^ The within-person variance is the overall residual variance in cognition that is not explained by the model. The initial status variance component is the variance of individuals’ intercepts about the intercept of the average person. The rate of change variance component is the variance of individual slopes about the slope of the average person.

× represents an interaction between the two factors; interactions are presented based on multiplicative interaction model

****p*≤0.001, ***p*≤0.01, **p*≤0.05

|  |  | **<65 Years of Age** | | | | **>65 Years of Age** | | | | | |
| --- | --- | --- | --- | --- | --- | --- | --- | --- | --- | --- | --- |
|  |  | **Men** | | **Women** | | **Men** | | | **Women** | |  |
|  |  | **β** | **95% CI** | **β** | **95% CI** | **β** | **95% CI** | | **β** | **95% CI** |  |
| *Baseline* | |  |  |  |  |  | |  |  |  |  |
|  | PGS | **1.27***** | **0.84, 1.71** | **1.56***** | **1.04, 2.08** | **.55*** | **-07, 1.02** | | **1.23***** | **0.52, 1.95** |  |
|  | Top Tertile | - | - | - | - | - | - | | - | - |  |
|  | Mid Tertile | -0.27 | -0.74, 0.20 | **0.69*** | **0.11, 1.27** | 0.05 | -0.51, 0.61 | | 0.31 | -0.44, 1.07 |  |
|  | Bottom Tertile | **-1.75***** | **-2.58, -0.93** | 0.88 | -0.07, 1.84 | 0.35 | -0.47, 1.18 | | 0.36 | -0.69, 1.41 |  |
|  | |  |  |  |  |  | |  |  |  |  |
|  | PGS × Top Tertile | - | - | - | - | - | - | | - | - |  |
|  | PGS × Mid Tertile | -0.02 | -0.51, 0.47 | 0.07 | -0.51, 0.65 | 0.39 | -0.14, 0.93 | | -0.10 | -0.86, 0.66 |  |
|  | PGS × Bottom Tertile | 0.33 | -0.43, 1.11 | **-0.99**** | **-1.91, -0.07** | 0.44 | -0.35, 1.23 | | 0.39 | -0.69, 1.46 |  |
|  |  |  |  |  |  |  |  | |  |  |  |
| *Rate of change* | |  |  |  |  |  | |  |  |  |  |
|  | PGS | 0.03 | -0.01, 0.07 | 0.01 | -0.04, 0.04 | 0.03 | -.02, 0.09 | | .08 | -.00, .17 |  |
|  | Top Tertile | - | - | - | - | - | - | | - | - |  |
|  | Mid Tertile | 0.04 | -0.00, 0.08 | -0.02 | -0.06, 0.02 | -0.03 | -0.09, 0.04 | | .01 | -0.08, 0.09 |  |
|  | Bottom Tertile | 0.02 | -0.05, 0.11 | 0.03 | -0.05, 0.11 | -0.01 | -0.11, 0.10 | | -.06 | -0.19, 0.07 |  |
|  |  |  |  |  |  |  |  | |  |  |  |
|  | PGS × Top Tertile | - | - | - | - | - | - | | - | - |  |
|  | PGS × Mid Tertile | -0.03 | -0.08, 0.01 | 0.00 | -0.04, 0.05 | -0.04 | -0.10, 0.03 | | -0.07 | -0.16, 0.02 |  |
|  | PGS × Bottom Tertile | -0.08 | -0.15, -.01 | 0.01 | -0.06, 0.10 | -0.00 | -0.09, 0.09 | | **-0.17*** | **-0.32, -0.02** |  |
|  |  |  |  |  |  |  |  | |  |  |  |
| *Variance ^a^* | |  |  |  |  |  | |  |  |  |  |
|  | Within-person | 0.05 | 0.03, 0.06 | 0.05 | 0.04, 0.06 | 0.03 | 0.02, 0.05 | | 0.04 | 0.03, 0.07 |  |
|  | In initial status | 15.80 | 14.70, 16.99 | 25.02 | 23.42, 26.72 | 12.85 | 11.74, 14.07 | | 21.09 | 19.39, 22.94 |  |
|  | In rate of change | 0.05 | -0.03, 0.14 | -0.02 | -0.13, 0.08 | -0.06 | -0.17, 0.04 | | 0.14 | -0.03, 0.31 |  |

**Supplementary Table 7. Longitudinal mixed models exploring the main effect of polygenic score for BMI and imputed subjective social status, and interaction between these two variables, in relation to BMI trajectories during the 12-year follow-up period, adjusted for imputed covariates**

CI, confidence intervals; PGS, polygenic score

The adjusted models were adjusted for 4 principal components to account for any ancestry differences in genetic structures that could bias the results, as well as; marital status, physical activity level, presence of longstanding limiting illness, self-reported health, depressive symptoms, and smoking status.

^a^ The within-person variance is the overall residual variance in cognition that is not explained by the model. The initial status variance component is the variance of individuals’ intercepts about the intercept of the average person. The rate of change variance component is the variance of individual slopes about the slope of the average person.

× represents an interaction between the two factors; interactions are presented based on multiplicative interaction model

****p*≤0.001, ***p*≤0.01, **p*≤0.05

|  |  | **<65 Years of Age** | | | | **>65 Years of Age** | | | | | |
| --- | --- | --- | --- | --- | --- | --- | --- | --- | --- | --- | --- |
|  |  | **Men** | | **Women** | | **Men** | | | **Women** | |  |
|  |  | **β** | **95% CI** | **β** | **95% CI** | **β** | **95% CI** | | **β** | **95% CI** |  |
| *Baseline* | |  |  |  |  |  | |  |  |  |  |
|  | PGS | **1.41***** | **1.12, 1.69** | **1.57***** | **1.24, 1.89** | **0.82***** | **0.42, 1.21** | | **1.33***** | **0.76, 1.91** |  |
|  | Top Tertile | - | - |  |  | - | - | | - | - |  |
|  | Mid Tertile | 0. 13 | -0.34, 0.57 | **0.56*** | **0.02, 1.09** | **-0.64*** | **-1.18, -0.10** | | **0.79*** | **0.09, 1.51** |  |
|  | Bottom Tertile | -0.43 | -0.91, 0.08 | **0.61*** | **0.05, 1.18** | -0.34 | -0.87, 0.19 | | **0.86*** | **0.19, 1.53** |  |
|  | |  |  |  |  |  | |  |  |  |  |
|  | PGS × Top Tertile | - | - | - | - | - | - | | - | - |  |
|  | PGS × Mid Tertile | 0.15 | -0.30, 0.61 | 0.05 | -0.48, 0.58 | -0.05 | -0.57, 0.48 | | -0.24 | -0.97, 0.48 |  |
|  | PGS × Bottom Tertile | **-0.66**** | **-1.14, -0.18** | -0.21 | -0.76, 0.33 | 0.23 | -0.28, 0.74 | | -0.17 | -0.83, 0.50 |  |
|  |  |  |  |  |  |  |  | |  |  |  |
| *Rate of change* | |  |  |  |  |  | |  |  |  |  |
|  | PGS | -.00 | -0.03, 0.02 | 0.01 | -0.01, 0.04 | 0.01 | -0.03, 0.06 | | -0.01 | -0.09, 0.06 |  |
|  | Top Tertile | **-** | - |  |  | - | - | | - | - |  |
|  | Mid Tertile | 0.04 | -0.01, 0.08 | 0.03 | -0.02, 0.07 | 0.05 | -0.01, 0.12 | | -0.01 | -0.09, 0.07 |  |
|  | Bottom Tertile | 0.03 | -0.01, 0.08 | 0.03 | -0.02, 0.08 | 0.06 | -0.01, 0.12 | | -0.04 | -012, 0.03 |  |
|  |  |  |  |  |  |  |  | |  |  |  |
|  | PGS × Top Tertile | - | - | - | - | - | - | | - | - |  |
|  | PGS × Mid Tertile | 0.00 | -0.04, 0.05 | -0.04 | -0.08, 0.01 | 0.00 | -0.06, 0.07 | | 0.06 | -0.03, 0.15 |  |
|  | PGS × Bottom Tertile | 0.02 | -0.01, 0.08 | 0.02 | -0.03, 0.06 | -0.03 | -0.09, 0.03 | | 0.04 | -0.05, 0.12 |  |
|  |  |  |  |  |  |  |  | |  |  |  |
| *Variance ^a^* | |  |  |  |  |  | |  |  |  |  |
|  | Within-person | 0.04 | 0.03, 0.06 | 0.05 | .04, 0.06 | .03 | 0.02, 0.05 | | .04 | 0.03, 0.07 |  |
|  | In initial status | 15.8 | 14.74, 17.04 | 25.22 | 23.48, 26.79 | 12.79 | 11.69, 14.01 | | 20.95 | 19.26, 22.80 |  |
|  | In rate of change | 0.05 | -0.02, 0.14 | -0.03 | -0.13, 0.08 | -.06 | -0.16, 0.05 | | 0.15 | -0.01, 0.32 |  |

**Supplementary Table 8. Longitudinal mixed models exploring the main effect of polygenic score for BMI and imputed income, and interaction between these two variables, in relation to BMI trajectories during the 12-year follow-up period, adjusted for imputed covariates**

CI, confidence intervals; PGS, polygenic score

The adjusted models were adjusted for 4 principal components to account for any ancestry differences in genetic structures that could bias the results, as well as; marital status, physical activity level, presence of longstanding limiting illness, self-reported health, depressive symptoms, and smoking status.

^a^ The within-person variance is the overall residual variance in cognition that is not explained by the model. The initial status variance component is the variance of individuals’ intercepts about the intercept of the average person. The rate of change variance component is the variance of individual slopes about the slope of the average person.

× represents an interaction between the two factors; interactions are presented based on multiplicative interaction model

****p*≤0.001, ***p*≤0.01, **p*≤0.05
